# Diagnostic yield of genetic testing in a multinational heterogeneous cohort of 2088 DCM patients

**DOI:** 10.3389/fcvm.2023.1254272

**Published:** 2023-09-19

**Authors:** Krista Heliö, Marcos Cicerchia, Julie Hathaway, Johanna Tommiska, Johanna Huusko, Inka Saarinen, Lotta Koskinen, Mikko Muona, Ville Kytölä, Janica Djupsjöbacka, Massimiliano Gentile, Pertteli Salmenperä, Tero-Pekka Alastalo, Christian Steinberg, Tiina Heliö, Jussi Paananen, Samuel Myllykangas, Juha Koskenvuo

**Affiliations:** ^1^ Heart and Lung Center, ERN GUARD-Heart Center, University of Helsinki and Helsinki University Central Hospital, Helsinki, Finland; ^2^ Blueprint Genetics, A Quest Diagnostics Company, Espoo, Finland; ^3^ Blueprint Genetics, A Quest Diagnostics Company, Seattle, USA; ^4^ Quebec Heart and Lung Institute, Laval University, Quebec, Canada

**Keywords:** dilated cardiomyopathy, cardiomyopathy, genetic testing, next generation sequencing, diagnostic yield

## Abstract

**Background:**

Familial dilated cardiomyopathy (DCM) causes heart failure and may lead to heart transplantation. DCM is typically a monogenic disorder with autosomal dominant inheritance. Currently disease-causing variants have been reported in over 60 genes that encode proteins in sarcomeres, nuclear lamina, desmosomes, cytoskeleton, and mitochondria. Over half of the patients undergoing comprehensive genetic testing are left without a molecular diagnosis even when patient selection follows strict DCM criteria.

**Methods and results:**

This study was a retrospective review of patients referred for genetic testing at Blueprint Genetics due to suspected inherited DCM. Next generation sequencing panels included 23–316 genes associated with cardiomyopathies and other monogenic cardiac diseases. Variants were considered diagnostic if classified as pathogenic (P) or likely pathogenic (LP). Of the 2,088 patients 514 (24.6%) obtained a molecular diagnosis; 534 LP/P variants were observed across 45 genes, 2.7% (14/514) had two diagnostic variants in dominant genes. Nine copy number variants were identified: two multigene and seven intragenic. Diagnostic variants were observed most often in *TTN* (45.3%), *DSP* (6.7%), *LMNA* (6.7%), and *MYH7* (5.2%). Clinical characteristics independently associated with molecular diagnosis were: a lower age at diagnosis, family history of DCM, paroxysmal atrial fibrillation, absence of left bundle branch block, and the presence of an implantable cardioverter-defibrillator.

**Conclusions:**

Panel testing provides good diagnostic yield in patients with clinically suspected DCM. Causative variants were identified in 45 genes. In minority, two diagnostic variants were observed in dominant genes. Our results support the use of genetic panels in clinical settings in DCM patients with suspected genetic etiology.

## Introduction

Familial dilated cardiomyopathy (DCM) is a significant cause of heart failure and heart transplantation. DCM is typically a monogenic disorder with autosomal dominant (AD) inheritance but autosomal *de novo*, recessive, X-linked, and mitochondrial patterns have also been observed (1, 2). Although genetic testing is recommended in familial DCM, it is not routinely used in all centers (3, 4). Identifying the underlying cause of DCM helps recognize family members at risk. Patients with a diagnostic variant may have a worse prognosis (5, 6).

The genetic etiology of DCM is more heterogenous than in other cardiomyopathies (7). Disease-causing variants have been identified in over 60 genes that encode a variety of proteins in sarcomeres, nuclear lamina, desmosomes, cytoskeleton, and mitochondria (1, 2). Truncating titin variants (*TTN*tv) are the most common genetic cause of DCM, and other common genes include *LMNA*, *MYH7*, *RBM20*, *TNNT2*, *TPM1*, *FLNC*, *DSP*, and *DES* (1, 8, 9). Currently over half of the patients undergoing comprehensive genetic testing are left without a molecular diagnosis even when patient selection follows strict DCM criteria (10–15).

We aimed to evaluate the diagnostic yield of genetic testing in a real-life setting in patients with a clinical suspicion of DCM, referred for genetic testing from multiple centers around the world. We present the genes in which diagnostic variants were identified and the clinical variables that influenced the likelihood of observing a diagnostic variant.

## Methods

### Participants

The study comprised 2,088 patients with clinically suspected inherited DCM. Inclusion criteria were (1) clinically suspected DCM by referring healthcare provider and (2) panel testing conducted at Blueprint Genetics laboratory. DCM diagnostic criteria were not used as inclusion criteria. Patients with only deletion/duplication analysis conducted were excluded. The patients were presumed to be affected and unrelated. Patient demographic, and all clinical data came from requisition forms completed by the ordering clinician.

### Genetic testing

Patients underwent testing as ordered by their healthcare provider aiming to find genetic cause for DCM. The panels included 23–316 genes associated with cardiomyopathies and other monogenic cardiac diseases.

Oligonucleotide-selective sequencing (OS-Seq™) NGS (next generation sequencing) method on the NextSeq™ sequencing system (Illumina) was used to analyze 637 (30.5%) participants. An in-house tailored Integrated DNA Technologies based whole-exome platform, or TWIST based clinical exome platform run on the NovaSeq™ sequencing system (Illumina) was used to analyze 501 (24.0%) and 950 patients (45.5%), respectively. Mitochondrial DNA was analyzed in 804 patients (38.5%). Mean sequencing coverage at 20× was 99.95% of target nucleotides and in all included cases at least 98.0% coverage at 20× was reached. All protein-coding exons of the genes on the panels and 20 base pairs (bp) inside each intron/exon boundary were included in the target nucleotides. Later versions of the panels were adjusted by including non-coding variants (promoter region, 5′ or 3′ untranslated regions) and oligonucleotides targeting deep-intronic variants (≥20 bp from the intron/exon boundary) that have been reported as disease causing and associating with cardiomyopathy or arrhythmias. The sequence variant analysis pipeline has been validated in a CLIA (Clinical Laboratory Improvement Amendments)- and CAP (College of American Pathologists)-accredited Blueprint Genetics diagnostic laboratory.

Bi-directional Sanger sequencing confirmed likely pathogenic (LP) and pathogenic (P) variants when quality criteria for a true positive call were not met. The quality criteria included a variant call quality score, genomic location of the variant, sequence content, and integrative genomics viewer visual analysis. These criteria were based on the outcome of an internal validation performed in the CLIA- and CAP-accredited Blueprint Genetics laboratory.

### Copy number variant analysis

Copy number variant (CNV) analysis was performed for 1,504 patients (72.0%) from the NGS data using a bioinformatic pipeline including a CNVkit and an in-house developed proprietary technology. Either quantitative polymerase chain reaction (qPCR) technology or digital droplet PCR (dPCR) were used to confirm CNVs. The CNV analysis pipeline has been validated in the CLIA- and CAP-accredited Blueprint Genetics laboratory.

### Interpretation of test results

The Blueprint Genetics classification scheme was used for variant classification. The classification scheme was based on and followed the American College of Medical Genetics and Genomics/Association for Molecular Pathology (ACMG/AMP) guidelines (16). To achieve a LP/P variant status, multiple independent lines of evidence must be met. A molecular diagnosis was defined as an LP/P variant consistent with the patient's reported phenotype and with known associated disease inheritance. Variants were defined as diagnostic if classified as LP/P. In *TTN*, only *TTN*tv were considered as LP/P and only when expression pattern supported pathogenicity. Consensus splice site variants in *TTN* were considered disease causing only when expected to cause out-of-frame transcript (out-of-frame exon or out-of-frame cryptic splice in relation to high PSI exon). The 10% removal rule of total protein length was not considered as a strong enough criterium for loss-of-function in case of in-frame deletion because variable length *TTN* transcripts have been shown biologically relevant (17).

### Statistical analysis

To compare groups, Fisher's exact or Chi-Square tests were used for categorical variables and unpaired T-test for normally distributed continuous variables. *P*-value <0.05 was considered statistically significant.

## Results

This study comprised 2,088 patients with clinically suspected inherited DCM. Over half were males (63%, *n* = 1,308). The mean age at the time of genetic testing was 46.2 years. Patient characteristics and clinical variables are outlined in Table 1.

**Table 1:** Patient demographic and clinical variables.

	All	Non-diagnostic	Diagnostic	*p*-value
	*N* = 2,088	*N* = 1,574	*N* = 514	
Males	1,308 (62.6%)	993 (63.1%)	315 (61.3%)	
Females	780 (37.4%)	581 (36.9%)	199 (38.7%)	
Age at primary diagnosis (years)	40.6 ± 18.3	41.5 ± 18.5	37.9 ± 17.3	0.007
Affected family members	678/1,581 (42.9%)	465/1,176 (39.5%)	213/404 (52.7%)	<0.001
Previous genetic testing	173/1,533 (11.3%)	130/1,150 (11.3%)	43/383 (11.2%)	1.00
ICD	291/1,079 (27.0%)	200/812 (24.6%)	91/267 (34.1%)	0.003
PM	142/1,035 (13.7%)	115/789 (14.6%)	27/246 (11.0%)	0.168
Heart transplant	40/1,046 (3.8%)	28/790 (3.5%)	12/256 (4.7%)	0.453
Symptoms	*n* = 955	*n* = 707	*n* = 248	
No symptoms	194 (20.3%)	144 (20.4%)	50 (20.2%)	1.000
Presyncope	44 (4.6%)	31 (4.4%)	13 (5.2%)	0.598
Collapse	44 (4.6%)	34 (4.8%)	10 (4.0%)	0.726
Arrhythmias at stress	57 (6.0%)	36 (5.1%)	21 (8.5%)	0.062
Arrhythmias at rest	149 (15.6%)	100 (14.1%)	49 (19.8%)	0.042
Decreased exercise tolerance	475 (49.7%)	364 (51.5%)	111 (44.8%)	0.076
Resuscitated	71 (7.4%)	53 (7.5%)	18 (7.3%)	1.00
Confirmed arrhythmias	*n* = 899	*n* = 669	*n* = 230	
No arrhythmias	526 (58.5%)	410 (61.3%)	116 (50.4%)	0.005
Asystole	4 (0.4%)	2 (0.3%)	2 (0.9%)	0.271
Ventricular fibrillation	49 (5.5%)	34 (5.1%)	15 (6.5%)	0.403
Ventricular tachycardia >3 beats	163 (18.1%)	112 (16.7%)	51 (22.2%)	0.074
Chronic atrial fibrillation	62 (6.9%)	42 (6.3%)	20 (8.7%)	0.228
Paroxysmal atrial fibrillation	85 (9.5%)	52 (7.8%)	33 (14.3%)	0.006
Conduction defects	*n* = 990	*n* = 760	*n* = 230	
No conduction defects	579 (58.5%)	420 (55.3%)	159 (69.1%)	<0.001
AVB1	76 (7.7%)	64 (8.4%)	12 (5.2%)	0.121
AVB2	7 (0.7%)	4 (0.5%)	3 (1.3%)	0.207
AVB3	29 (2.9%)	22 (2.9%)	7 (3.0%)	0.827
LBBB	238 (24.0%)	208 (27.4%)	30 (13.0%)	<0.001
LAHB	46 (4.6%)	38 (5.0%)	8 (3.5%)	0.378
RBBB	25 (2.5%)	22 (2.9%)	3 (1.3%)	0.233

Non-diagnostic for patients with no observed LP/P variant, Diagnostic for patients with observed LP/P variant, ICD for implantable cardioverter-defibrillator, PM for pacemaker, AVB for atrioventricular block, LBBB for left bundle branch block, LAHB for left anterior hemiblock, RBBB for right bundle branch block. *P*-values were calculated comparing the non-diagnostic group to the diagnostic group.

### Diagnostic yield and variant profile

Altogether 534 disease-causing variants (209 P and 325 LP) were identified across 45 genes (Supplementary S1). Of the 2,088 patients, a diagnostic variant was observed in 24.6% (*n* = 514). Diagnostic yield was 24.1% (315/1,308) for males, 25.5% (199/780) for females, 24.2% in infants (23/95, ≤1 years), 22.8% in all pediatric patients (50/219, <18 years), and 24.9% in adults (465/1,869, ≥18 years).

The panels included 23–50 genes in 10.7%, 51–100 genes in 32.9%, and 101–316 genes in 56.4%. The diagnostic yields were 26.5%, 24.3%, and 24.5% respectively for the panels by size groups. 173 patients had previously undergone genetic testing without a molecular diagnosis; 24.9% (43/173) of them obtained one after using the panels included in this study.

Diagnostic variants were detected most often in *TTN* (45.3%) followed by *DSP* (6.7%), *LMNA* (6.7%), *MYH7* (5.2%), *TNNT2* (4.5%), *FLNC* (4.1%), *RBM20* (3.6%), and *BAG3* (2.2%) (Table 2). The remaining 37 genes had ten or fewer diagnostic variants each. Molecular diagnosis was consistent with AD inheritance in 93.2% (*n* = 479), followed by autosomal recessive (3.5%, *n* = 18), X-linked (2.3%, *n* = 12), and mitochondrial (1.0%, *n* = 5). Fourteen patients (2.7%) had two diagnostic variants in dominant genes (*TTN *+ *TTN* in three patients, *TTN *+ *DSP* in three patients, *TTN *+ *RBM20* in two patients, and the following in one patient each: *CASZ1 *+ *MT-TL1*, *TTN *+ *DSG2*, *DSP *+ *FLNC*, *LMNA *+ *MYBPC3*, *MYH7 *+ *PKP2*, and *TNNT2 *+ *DSP*). In an enrichment analysis of *PRDM16* loss-of-function (LoF) variants were significantly enriched in our cohort (3/2,088) when compared to GnomAD reference population (4/124,635, *p* < 0.0001), with an odds ratio of 44.8 (95% CI, 10.0, 200.4).

**Table 2:** Distribution of LP/P variants by gene.

Gene	Number of variants (*n* = 534)	Percentage of all variants observed (%)
TTN	242	45.3
DSP	36	6.7
LMNA	36	6.7
MYH7	28	5.2
TNNT2	24	4.5
FLNC	22	4.1
RBM20	19	3.6
BAG3	12	2.2
NRAP	10	1.9
DSG2	9	1.7
SCN5A	6	1.1
DES	5	0.9
DMD	5	0.9
MT-TL1	5	0.9
PKP2	5	0.9
ALMS1	5	0.7
MYBPC3	4	0.7
PLN	4	0.7
TBX20	4	0.7
TPM1	4	0.7
EMD	3	0.6
MYL2	3	0.6
PRDM16	3	0.6
RYR2	3	0.6
GATA4	2	0.4
GLB1	2	0.4
LAMP2	2	0.4
LMOD2	2	0.4
TAB2	2	0.4
TNNI3	2	0.4
APOA1	1	0.2
CASZ1	1	0.2
FKRP	1	0.2
GATA6	1	0.2
GCOM1/MYZAP	1	0.2
GLA	1	0.2
MT-TK	1	0.2
NDUFVK, SMCHD1	1	0.2
NEXN	1	0.2
NKX2-5	1	0.2
PLEKHM2	1	0.2
SLC25A4	1	0.2
TAZ	1	0.2
TNNC1	1	0.2
TTR	1	0.2
Undisclosed gene	10	1.9

Distribution of LP/P variants by gene as percentage of all variants observed (*n* = 534).

### CNV

Nine diagnostic CNVs were identified: two multigene and seven intragenic. One patient had a 13.7 Mb multigene deletion, and one patient had a 3.7 Mb deletion involving the whole *GATA4* gene. Two CNVs were observed in *DMD:* a deletion encompassing exons 48–51 (*DMD* c.(6,912 + 1_6913-1)_(7,542 + 1_7543-1)del) and a duplication encompassing exons 19–37 (*DMD* c.(2,292 + 1_2293-1)-c.(5,325 + 1_5326-1)dup). One patient had a 3 kb deletion-insertion in *LMNA* c.437_514-772delinsAGTTCTGAGCACTGCTCTCACTGCT. A 51.9 kb deletion in *TBX20* was observed. One patient with ventricular fibrillation, dilated LV, and features compatible with left ventricular non-compaction cardiomyopathy (LVNC) had the common pathogenic *RYR2* exon 3 deletion, c.(168 + 1_169-1)_(273 + 1_274-1)del, as a *de novo* variant. Additionally, two single exon deletions were observed: one in exon 8 of *PKP2* c.(1,688 + 1_1689-1)_(1,806 + 1_1807-1)del and one in *DSP* c.(?_-341)_(170_?)del (NM_004415.3) encompassing exon 1.

### Other findings

Of the patients with diagnostic variants, 1.6% (8/514) had a variant in genes (*DMD* and *EMD*) associated with neuromuscular diseases. Two variants were observed in genes (*GLA* and *TTR*) associated with diseases that have targeted therapies.

Nine patients had variants in genes associated with arrhythmias, six in *SCN5A* and three in *RYR2*. All patients with *SCN5A* variants had DCM according to imaging findings, five had familial DCM, and five had a significant amount of ventricular extrasystoles (VES). Two had been resuscitated from sudden cardiac arrest and two had family history of sudden cardiac death (SCD) at young age. Two of the three patients with *RYR2* variants had ventricular fibrillation and severe DCM which could be explained by post-arrest changes. One patient with *RYR2* variant had significant arrhythmias at stress test fitting to catecholaminergic polymorphic ventricular tachycardia (CPVT), and a significant family history for SCD. Of the patients with diagnostic variants, 15.8% (81/514) had a variant in genes that have been associated with increased risk of ventricular arrhythmias (*LMNA, RBM20, FLNC,* and *PLN*) (18). Four patients had a diagnostic variant in *MYBPC3*, which is predominantly associated with hypertrophic cardiomyopathy (19). No imaging findings were available on these four patients to assess the wall thickness.

### Clinical characteristics and diagnostic yield

A family history of DCM increased the likelihood of identifying LP/P variant (*p* < 0.001): a diagnostic variant was observed more often in those with affected family members (31.4%, 213/678) than in those with a reportedly sporadic disease (21.2%, 191/903).

**Figure 1:**
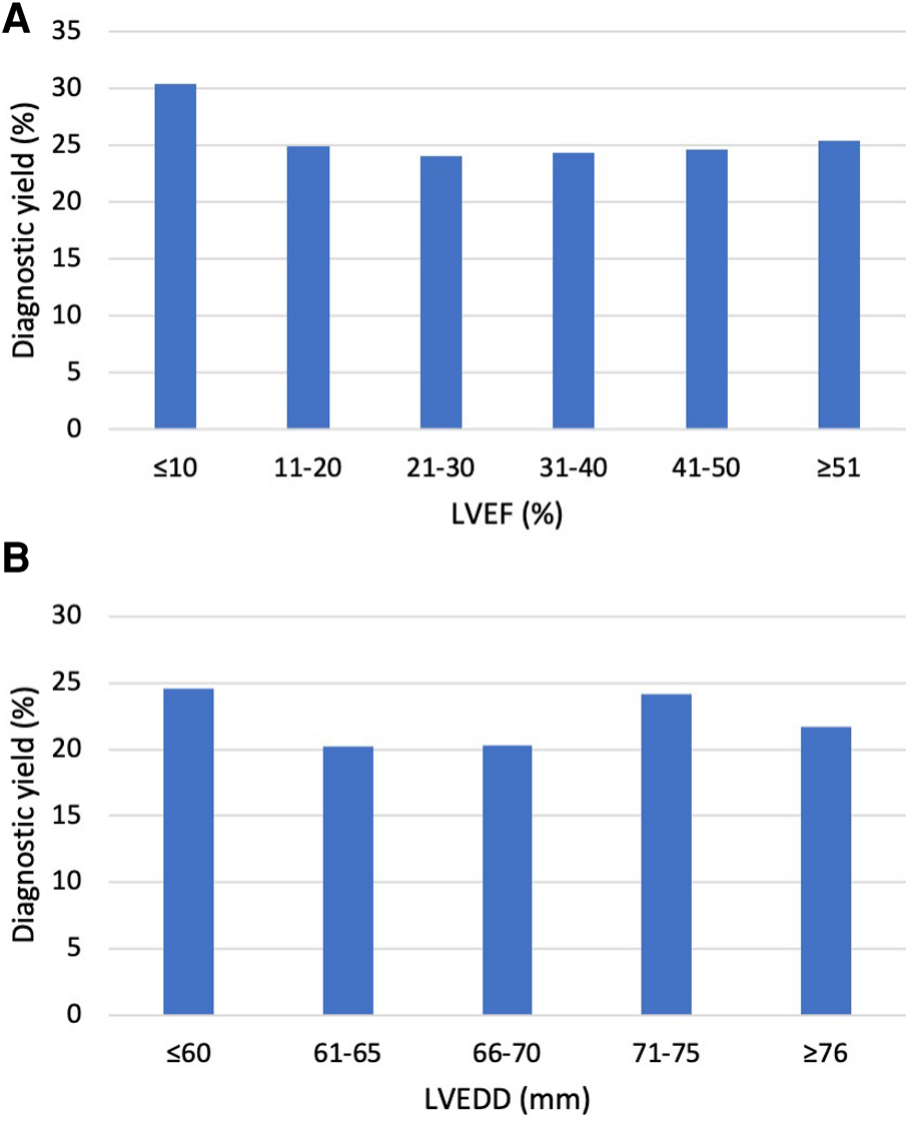
Diagnostic yield by echocardiographic findings in adult patients. (**A**) Diagnostic yield by LVEF in patients ≥18 years. The Y-axis indicates the percentage of observed LP/P variants, and the X-axis indicates left ventricular ejection fraction (LVEF). The diagnostic yield was similar in all groups (*p* = 0.97). (**B**) Diagnostic yield by left ventricular end-diastolic diameter (LVEDD) in patients ≥18 years. The Y-axis indicates the percentage of observed LP/P variants, and the X-axis indicates LVEDD in millimeters. The diagnostic yield was similar in all groups (*p* = 0.82).

Left ventricular end-diastolic diameter (LVEDD) was reported in 709 patients, 157 of them had a diagnostic variant. Left ventricular ejection fraction (LVEF) was reported in 1,108 patients, 274 of them had a diagnostic variant. There was no significant difference (*p* = 0.669) in EF in patients with (29.5 ± 12.6%) and without (29.9 ± 12.4%) a molecular diagnosis. Mean LVEDD was similar in the two groups: 65.9 ± 9.2 mm in those with a diagnostic variant and 66.1 ± 8.7 mm in those without (*p* = 0.819). In patients over the age of 18, neither larger LVEDD nor lower LVEF affected the likelihood of observing a diagnostic variant (Figure 1). Echocardiographic findings were similar in patients with a variant in *TTN, DSP, LMNA, FLNC, MYH7,* and *TNNT2* (Table 3).

**Table 3:** Echocardiographic findings by gene in adult patients.

	TTN	DSP	LMNA	FLNC	MYH7	TNNT2
LVEDD (mm)						
n	88	14	10	8	5	5
Median	66.0	59.0	57.5	69.0	73.0	71.0
IQR	61.0–71.5	57.0–68.0	50.0–62.0	62.0–79.0	69.0–74.0	62.0–85.0
LVEF (%)						
n	156	18	17	10	11	9
Median	28.0	25.0	30.0	26.0	39.0	23.0
IQR	20.0–35.0	23.0–40.0	29.0–38.0	25.0–36.0	20.0–43.5	20.0–33.0

The echocardiographic findings of patients ≥18 years with diagnostic variants in *TTN*, *DSP*, *LMNA*, *FLNC*, *MYH7*, and *TNNT2*. LVEDD for left ventricular end-diastolic diameter, LVEF for left ventricular ejection fraction, IQR for inter quartile range.

### Symptoms and arrhythmias

Patients with any clinically confirmed arrhythmias reported by their ordering provider had diagnostic variants more often when compared to those with no confirmed arrhythmias (30.6% [114/373] vs. 22.1% [116/526], *p* = 0.005). A diagnostic variant was observed more frequently in patients who had arrhythmias at rest than in those without (32.9% [49/149] vs. 24.7% [199/806], *p* = 0.042).

An LP/P variant was observed more often in those with reported paroxysmal atrial fibrillation (AF) (38.8% [33/85] vs. 24.2% [197/814], *p* = 0.006). Patients with an implantable cardioverter-defibrillator (ICD) were more likely to have a diagnostic variant (31.3% [91/291] vs. 22.3% [176/788], *p* = 0.003). The absence of symptoms, arrhythmias at stress, presyncope, syncope, decreased exercise tolerance, asystole, ventricular fibrillation, ventricular tachycardia >3 beats, resuscitation, chronic AF, pacemaker, and heart transplantation were equally common in the diagnostic and non-diagnostic groups.

### Conduction defects

A diagnostic variant was observed more often in the group with no reported conduction defects (27.5% [159/579] vs. 17.3%, [71/411], *p* < 0.001). The absence of left bundle branch block (LBBB) was more common in those with a diagnostic variant (26.6% [200/752] vs. 12.6% [30/238], *p* < 0.001). Atrioventricular blocks (AVBs), left anterior hemiblock (LAHB), and right bundle branch block (RBBB) were equally common in the diagnostic and non-diagnostic groups.

### Age

**Figure 2:**
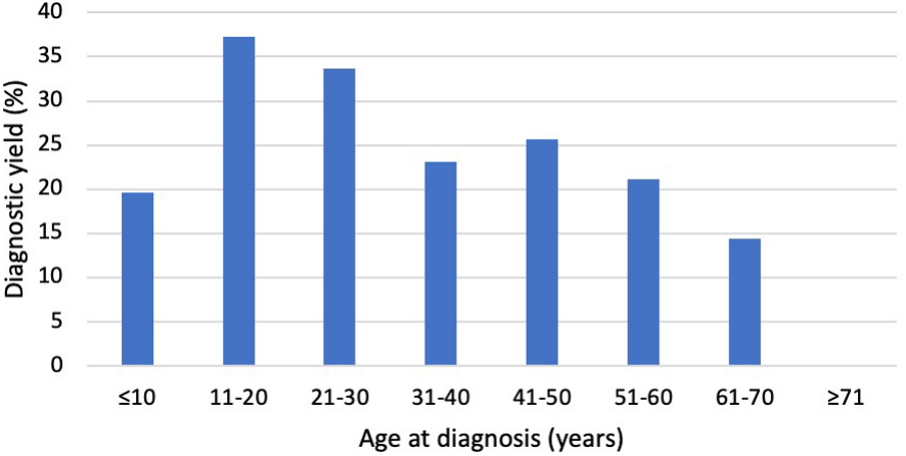
Diagnostic yield by age at diagnosis. The Y-axis indicates the percentage of patients with LP/P variants, and the X-axis indicates the age at diagnosis. The diagnostic yield was greatest in patients aged 11−20 and 21−30 years. None of the patients diagnosed after the age of 70 had LP/P variants.

The age at primary diagnosis was reported in 49.5% of the cohort (1,033/2,088). The mean age at diagnosis was lower (*p* = 0.007) in patients with a diagnostic variant (37.9 ± 17.3) when compared to those with no diagnostic finding (41.5 ± 18.5). The diagnostic yield was highest in patients aged 11–20 years (37.3%) at the time of DCM diagnosis, whereas none of the patients who received the DCM diagnosis after the age of 70 obtained a molecular diagnosis (Figure 2).

## Discussion

A diagnostic variant was found in 24.6% of the 2,088 patients. The results in this heterogenous population are comparable to other studies, even though in previous studies the cohorts were selected using stringent DCM diagnostic criteria. Our cohort was selected based on suspected DCM and no supporting clinical data were used in patient selection. This heterogenous cohort is comparable to the real-life patients with suspected DCM referred for genetic testing.

Previous studies with patients fulfilling DCM diagnostic criteria have observed an overall diagnostic yield of 19%–47% (11, 12, 15, 20). In the recent ESC EURObservational Research Programme (EORP) Cardiomyopathy Registry study 33% of the patients had a diagnostic variant (13). The study included patients from 18 countries and strict DCM criteria were used as an inclusion criterion (13). The slightly higher overall diagnostic yield in the EORP study could be partly explained by the results of the genetic testing not being strictly controlled; the pathogenicity of the variant was self-reported by the investigators at the time of inclusion (13).

We observed that positive family history, lower age at diagnosis, paroxysmal AF, arrhythmias at rest, and the absence of LBBB were independently associated with molecular diagnosis. Previously observed factors associated with diagnostic test results have been skeletal myopathy, family history of DCM, low voltage on ECG, absence of hypertension, and absence of LBBB (6, 20, 21). Diagnostic yield has been consistently higher in those with a familial disease (36%–64%) when compared to sporadic DCM (13%–36%) (11, 12, 15, 20, 22). Genetic testing has been generally recommended in familial DCM, but taken together, these findings suggest that this recommendation might be too strict as LP/P variants have been identified in up to a third of those with no affected family members (4, 11, 12, 15, 22).

Even though LBBB is typical in patients with *LMNA* variants*,* it is rarer in other genetic etiologies, such as those with *TTN*tv (23). As the number of patients with *LMNA* variants was relatively low, the tendency for LBBB was not reflected in the overall association. AF has been associated with diagnostic test results, but the subtype has not been defined (5, 6, 20). We observed chronic AF to be equally common in the groups with and without a diagnostic variant whereas paroxysmal AF was more common in those with a positive genetic test.

Most diagnostic variants were observed in *TTN,* followed by *DSP*, *LMNA*, *MYH7*, *TNNT2*, and *FLNC*. The distribution of variants was similar when compared to previous reports (6, 11, 15). In our cohort, two diagnostic variants in dominant genes were observed in 2.7%. This is consistent with the EORP registry study and the recent cohort study by Stroeks et al., in both studies 2.3% of the patients had at least two diagnostic variants (13, 24). Stroeks et al. observed that patients with multiple variants did neither appear to have more severe disease nor an earlier disease onset (24). By contrast, some case studies have reported an earlier onset and more severe clinical course in patients carrying multiple variants in DCM-associated genes (25–28). As there are no larger studies of patients with multiple variants, it is still not known, how carrying more than one disease-causing variant affects the patient's clinical course. In our cohort, only 14 patients carried more than one diagnostic variant and the patients had different variant combinations. Due to small sample size as well as limited clinical data, we were not able to carry further analysis concerning the possible severity of the phenotype. Even though future research is still needed to assess how having more than one variant may affect the severity and prognosis of the disease, the reported prevalence of more than one variant in multiple cohort studies highlights the importance of comprehensive genetic testing. Regardless of the possible effect of multiple variants on the phenotype, the identification of all pathogenic variants contributing to the disease affects future family screening and cascade genetic testing.

We identified diagnostic variants also in genes less frequently associated with DCM. Diagnostic variants in *PRDM16* were observed in three patients with DCM, two of them had also features of LVNC. Deletions, missense, and frameshift variants in *PRDM16* have been associated mostly with LVNC but also with DCM (29, 30). *PRDM16* is predicted to be intolerant to LoF with a maximal probability of loss-of-function intolerance (pLI) value of 1.00 in gnomAD. Additionally, in our enrichment analysis LoF variants in *PRDM16* were significantly rarer in the gnomAD reference population when compared to our cohort.

One patient with severe left ventricular dysfunction (LVEF <20%) had a diagnostic variant in *PLEKHM2*. To our knowledge this patient represents the third family with homozygous/compound heterozygous truncation of *PLEKHM2*. In previous studies the patients with *PLEKHM2* variants presented with DCM and features of LVNC, and a family history of SCD (31, 32).

One patient with LP/P variants in *CASZ1* and *MT-TL1* presented with DCM, short stature, bilateral hearing-loss, low muscle mass, bilateral ptosis, diabetes, and multiorgan dysfunction. Heterozygous LoF variants in *CASZ1* have been associated with DCM (33–35). *CASZ1* is also involved in 1p36 deletion syndrome, which is characterized with dysmorphic facial features, intellectual disability, developmental delay, hearing loss, seizures, cardiomyopathy, and cardiovascular malformations (36). The pLI score of *CASZ1* in gnomAD is 1.00 suggesting that this gene is intolerant for LoF variation. According to the available evidence, LoF can be considered an established disease-mechanism in this gene. One patient had a homozygous truncating variant in *GCOM1/MYZAP*. DCM caused by homozygous *GCOM1/MYZAP* variants have been reported by us and one other group (37, 38). The GRINL1A complex transcription unit (CTU) contains the downstream gene *POLRM2* and the upstream gene *MYZAP,* as well as the *GCOM1* combined gene that uses exons from the upstream and downstream genes as well as its own exons (39). Due to the complexity of the GRINL1A CTU, a variant in *GCOM1* may also affect *MYZAP* transcripts, thus, variants can be called into different genes within the CTU. Relying on genotype tissue expression data, the affected gene should be called *MYZAP*.

Consistent with previous studies, most LP/P variants followed an AD inheritance. We observed LP/P variants across 45 genes. Currently over 60 genes have been associated with DCM. Due to lack of clinical data and varying variant interpretation, some of these 60 genes lack reliable evidence (10). Recent studies have aimed to re-assess the genes associated with monogenic DCM (14). Mazzarotto et al. defined 12 genes with strong association with DCM (*TTN, LMNA, MYH7*, *TNNT2*, *TPM1*, *DSP*, *VCL*, *BAG3*, *TNNC1*, *ACTC1*, *NEXN*, and *PLN* (14). Verdonschot et al. observed that almost 90% of their LP/P variants were in these 12 genes and additional 1.2% of the LP/P variants were in *RBM20* and *FLNC* (20). Stroeks et al. observed that using a gene panel including these 14 genes, an LP/P variant was found in 16.9% of the patients, whereas an extended panel with 48 genes resulted in a diagnostic yield of 17.8% (5). In our cohort 388 (72.7%) LP/P variants were observed in these 12 genes and additionally 41 variants were observed in *RBM20* and *FLNC*. If the patients in our cohort were tested with panels that included only these 14 genes, 19.7% (105/534) of the LP/P variants would have been missed. In our cohort, no variants were observed in *VCL* or *ACTC1.* This could be partly explained by the observation that *VCL* and *ACTC1* are more prevalent in pediatric patients (14).

The reassessment of genes has been based on individuals who fulfil strict DCM diagnostic criteria. This does not completely reflect the real-life situation in the clinics as sometimes the phenotype might overlap with other cardiomyopathies. Assigning a specific phenotype might come down to the personal interpretation of the clinician and the imaging modalities available. Previous publications suggest that the number of genes in a panel could be reduced, however, due to difficulties in phenotyping the patient, a larger panel combining different cardiomyopathy genes might be necessary.

Patients with LP/P variants have a worse prognosis, and they experience more adverse events (6, 20). Genetic etiology may affect clinical decision making. In our cohort 16% of those with molecular diagnosis had a LP/P variant in *LMNA, RBM20, FLNC,* or *PLN*. Pathogenic variants in these genes have been associated with increased risk of sustained ventricular arrhythmias. The ESC guidelines recognize pathogenic variants in these genes as a risk factor in patients with DCM when considering ICD implantation (18).

## Limitations

Data were based on the requisition forms completed by the ordering provider. Complete data were not available on all patients. No DCM criteria were used to confirm the diagnosis. The main genes associated with DCM were evaluated in all patients, but all patients were not tested with the same panels. LP/P variants identified in 14 patients were incorporated to the overall diagnostic yield but were not disclosed in this study as they represent new genes or founder variants that will be published in the future. In ten of these fourteen patients, the variant was observed in the same gene.

## Conclusions

Comprehensive NGS panels provide high diagnostic yield in both pediatric and adult patients with suspected inherited DCM. Genetic etiology is relatively wide, and causative variants were observed in 45 genes. Majority had AD disease, but LP/P variants were also observed in genes associated with recessive, X-linked or mitochondrial diseases, and some had double diagnosis in dominant genes—all supporting the use of genetic panels.

## Data Availability

The datasets presented in this article are not readily available because of the need to maintain the privacy of the individuals who participated in the study and to comply with GDPR legislation. Requests to access the datasets should be directed to the corresponding author.
